# Flagella-dependent inhibition of biofilm formation by sub-inhibitory concentration of polymyxin B in *Vibrio cholerae*

**DOI:** 10.1371/journal.pone.0221431

**Published:** 2019-08-20

**Authors:** Sean Giacomucci, Candice Danabé-Nieto Cros, Xavier Perron, Annabelle Mathieu-Denoncourt, Marylise Duperthuy

**Affiliations:** Département de microbiologie, infectiologie et immunologie, Université de Montréal, Succ. Centre-ville, Montréal, Québec, Canada; Nitte University, INDIA

## Abstract

Biofilm formation is a common strategy used by bacteria in order to survive and persist in the environment. In *Vibrio cholerae* (*V*. *cholerae*), a Gram-negative pathogen responsible for the cholera disease, biofilm-like aggregates are important for the pathogenesis and disease transmission. Biofilm formation is initiated by the attachment of the bacteria to a surface, followed by maturation stages involving the formation of a biofilm matrix. In *V*. *cholerae*, flagella are essential for the initial step of biofilm formation, allowing the bacteria to swim and to detect a surface. In this study, we explored the effect of polymyxin B (PmB), a cationic bacterial antimicrobial peptide, on biofilm formation in pathogenic *V*. *cholerae* strains belonging to the O1 and O139 serotypes. We found that sub-inhibitory concentration of PmB induces a reduction of the biofilm formation by *V*. *cholerae* O1 and O139. Experiment on preformed biofilm demonstrated that the biofilm formation inhibition occurs at the initial step of biofilm formation, where the flagella are essential. We further characterize the effect of PmB on *V*. *cholerae* flagellation. Our results demonstrate that the flagellin expression is not reduced in presence of sub-inhibitory concentration of PmB. However, a decrease of the abundance of flagellin associated with the bacterial cells together with an increase in the secretome was observed. Electron microscopy observations also suggest that the abundance of aflagellated bacteria increases upon PmB supplementation. Finally, in agreement with the effect on the flagellation, a reduction of the bacterial motility is observed. Altogether, our results suggest that the PmB affect *V*. *cholerae* flagella resulting in a decrease of the motility and a compromised ability to form biofilm.

## Introduction

*Vibrio cholerae* is the causative agent of the cholera disease. Infection usually occurs by consumption of food or water contaminated with *V*. *cholerae*. There are two serogroups that can cause the cholera disease, *i*.*e*. O1 and O139 [1]. Inside the O1 serogroup, 2 distinct biotypes have been described: the Classical biotype was responsible for 6 pandemics, which occurred before 1961, and the El Tor biotype, which is responsible for the ongoing 7^th^ pandemic after displacing the Classical biotype. The serogroup O139 has been identified in the early 1990s during an epidemic in Asia and is genetically derived from an O1 El Tor biotype [2]. The main virulence factors of the O1 and O139 serogroups are the cholera toxin and the toxin co-regulated pilus, responsible for the massive fluid loss and dehydration characteristic of cholera [1]. The non-O1/non-O139 strains of *V*. *cholerae* are also responsible for diarrheal diseases, which are less severe than the O1 and O139 serotypes [3].

To survive in their environment, majority of the bacteria form biofilms. A biofilm is a microbial community embedded in a self-produced extracellular matrix. The bacteria in the biofilm are more resistant to the immune system, to antibiotic treatments and to osmotic, oxidative and acidic stresses [4, 5]. It has been estimated that around 80% of the bacterial infections are related to biofilms with a resistance up to 1,000 times higher in biofilm compared to planktonic lifestyle [4]. In *V*. *cholerae*, it has been established that biofilm forms in the human gut [6] and that the biofilm formation is induced by the adherence to the human intestinal cells [7]. In the environment, *V*. *cholerae* can form biofilm on biotic and abiotic surfaces, including the chitin of zooplankton [8]. This biofilm lifestyle in the environment is important for the persistence and survival between epidemic seasons but also drastically increases the infectivity of *V*. *cholerae* [9]. The biofilm matrix of *V*. *cholerae* is mainly composed of exopolysaccharides (VPS), whose synthesis is encoded by two *vps* operons (VpsI and VpsII). In addition, the matrix proteins RbmA, RbmC and Bap1 are key determinants of the 3 dimensional structure of the biofilm [10, 11]. Bap1 is also involved in antimicrobial peptide cross-resistance in *V*. *cholerae* [12]. The biofilm formation is tightly regulated by a complex network involving a positive regulation by two major transcriptional regulators, VpsR and VpsT [13, 14] and a negative regulation by the quorum-sensing master regulator HapR. In addition, the stringent response (44), the c-di-GMP signaling pathway [15] and other regulators such as the calcium-controlled negative regulator CarR [16] are also involved in the regulation of biofilm formation in *V*. *cholerae*.

Biofilm formation can be divided in 5 main stages: the reversible attachment of planktonic cells, the irreversible attachment, the early development with the production of the matrix components, the maturation with the formation of the 3D structure and the dispersion, which result in planktonic bacterial release. In *V*. *cholerae* O1, the flagella and the mannose-sensitive hemagglutinin (MSHA) pili are essential during the first stages of biofilm development. The flagella are involved in the swimming motility necessary for the bacteria to reach a surface. Then, the bacteria use the flagella and the MSHA pili synergistically to irreversibly attach to the surface [17]. Conversely, the MSHA pili is not required for *V*. *cholerae* O139 biofilm formation [18]. The flagella rotor is essential for both O1 and O139 strains in the transmission of the signal of the bacterial contact with a surface, which activates the expression of the *vpsI* and *vpsII* operons allowing the biofilm to enter the third stage of its development, *i*.*e*. the early development stage [16, 18].

The flagellum of *V*. *cholerae* is composed of 5 highly homologues flagellin subunits, FlaA, FlaB, FlaC, FlaD and FlaE, arranged in two *loci*, *i*.*e*., *flaAC* and *flaEDB [19]*. FlaA is the only flagellin essential for the filament synthesis and the motility in *V*. *cholerae*, the four others being dispensable [19, 20]. In addition, *V*. *cholerae* flagellum is coated by an outer membrane sheath [21]. Flagellum driven motility is not only essential for biofilm formation and for intestine colonization of mice [18, 22]. Several adhesion genes are co-regulated with the flagellum genes, whereas virulence genes are inversely regulated, including the cholera toxin and the toxin co-regulated pilus [23, 24].

Antimicrobial peptides (AMPs) are oligopeptides of 12 to 50 amino-acids, mainly cationic and amphiphilic, with antimicrobial or immunomodulatory properties [25, 26]. Because of their multiple intracellular and membrane targets in the bacterial cells, the development of resistance is expected to be limited [27]. Therefore, AMPs are considered as valuable candidates for food preservatives and alternatives therapeutic agents [28]. AMPs can have eukaryotic or prokaryotic origin [29]. In the case of a eukaryotic origin, they are commonly named as host defense peptides and represent essential molecular effectors of the innate immunity [26]. The AMPs secreted by bacteria, also known as bacteriocins, are involved in inter-bacterial competition in order to protect their niche against foreign bacteria [30]. The lethal mechanism of cationic AMPs usually involves electrostatic interactions with the bacterial cell wall, integration in the membrane and pore formation, leading to the loss of the periplasmic and cytoplasmic components and, ultimately, to the death of the cell. More recently, a role of the AMPs as signaling molecules modulating the virulence of bacterial pathogens has been proposed [31–34]. In *V*. *cholerae*, our previous studies have clearly established that virulence and antimicrobial resistance proteins associated to membrane vesicles are up-regulated in response to the presence of sub-inhibitory concentrations of AMPs [12, 35]. We demonstrated that the association of the biofilm matrix protein Bap1 to the membrane vesicles is increased in presence of sub-inhibitory concentration of polymyxin B (PmB), a cationic bacterial AMP produced by *Bacillus polymyxa*, leading to the cross-resistance with the human cathelicidin LL-37 [12]. In contrary to O1 classical strains, O1 El Tor strains and O139 strains are known to be resistant to PmB [36, 37] In this study, we have focused on the effect of sub-inhibitory concentration of PmB in the modulation of biofilm formation in *V*. *cholerae* O1 El Tor and O139. Using *V*. *cholerae* strains from both serogroups related to cholera disease, *i*.*e*. O1 and O139, we found that sub-inhibitory concentration of PmB can significantly impair *V*. *cholerae* biofilm formation during the initial adhesion step, by affecting the flagellar synthesis and shape.

## Materials and methods

### Bacterial strains and culture conditions

A1552 (O1, El Tor, Inaba) and MO10 (O139) *V*. *cholerae* strains have been isolated from a traveler from Peru who contracted cholera in 1992 [38] and during a cholera outbreak in India in 1992 [39, 40], respectively. Bacteria were grown in Luria-Bertani (LB) broth at 37°C with shaking. Then, overday cultures were grown from 1:50 diluted overnight culture. Both overnight and overday cultures are grown in LB medium under shaking condition at 120 RPM. When overday cultures reach optical density (OD_600nm_) of 0.3, final cultures are started with or without 25 μg/mL PmB (Polymyxin B sulfate, Millipore, CAS#1405-20-5 lot:1305RP30930) by diluting to a theoretical OD_600nm_ of 0.006 in 2 mL LB within 5 mL polystyrene tube (Falcon ref.: 352058) and incubated at 37°C under shaking condition (230 RPM). PmB working concentrations were defined as ¼ of minimal inhibitory concentration and were determined as sub-inhibitory concentration using the culture conditions described above.

### Determination of the minimal inhibitory concentration (MIC)

The MIC was determined using the culture condition described in the Bacterial strain and culture condition section. For the motility assay, MIC was determined on LB agar plate. Serial dilutions of PmB were tested ranging from 100 μg/mL to 6.25 μg/mL. Growth (OD) was monitored spectrophotometrically at 600 nm after 16 h at 37°C. MIC values are expressed as the lowest concentration that causes 100% growth inhibition (μg/mL).

### Flagellar motility assay

Overnight and overday cultures were performed as described above. Overday cultures in early-exponential phase were adjusted to OD_600nm_ = 0.3 then 3 μl were spotted on motility plates and incubated at 37°C. Motility plates were made from 0.5% NaCl LB and 0.3% (w/v) agar, with 25 μg/mL to 0.39 μg/mL PmB or without (PmB solution was replaced by ddH2O). Diameters of motility were measured after 12h, 24h and 48h. Results were done in technical duplicates and at least in biological triplicates.

### Transmitted electron microscopy

Samples from exponential growth phase cultures were washed in phosphate buffer and fixed in 2.5% glutaraldehyde prepared in cacodylic acid 100 mM pH 7.4 buffer. Fifty microliters of fixed cells were placed on a 200 Mesh hexagonal slim bar grids covered with formvar and carbon. Samples were washed 3 times with 50 mM cacodylate acid saccharose 3% pH 7.4 buffer. Then samples were labeled with 1% phosphotungstic acid for 2 seconds and dried with blotting paper. Acquisitions were realized with transmitted electron microscope (TEM Hitachi H-7100 and AMT XR111 camera) at 75 kV and captured by digital camera (AMT XR-111, Advanced Microscopy Techniques).

### Biofilm assay

Biofilm quantifications were performed after 24h growth in LB medium with or without 25 μg/mL of PmB. Final OD_600nm_ was measured using the planktonic fraction before emptying and washing the tubes with ultrapure water. Biofilm quantification was performed using the crystal violet method [41]. Briefly, tubes were stained with 0.1% crystal violet (Alfa Aesar, #22866) and rinsed twice with ultrapure water. Finally, biofilms were dissolved in 33% acetic acid after 1 hour drying. Optical densities were measured in technical duplicates within, at least, biological triplicates. For biofilm kinetics measurements, series of tubes were filled with a common mix of the proper equilibrated overday culture and PmB solution or sterile water and biofilm quantification as describe in the Bacterial strains and culture conditions section.

### Flow cytometry

Samples were taken from final culture tube after 3 hours incubation at 37°C 230 RPM, which corresponded to an OD_600nm_ = 1, and diluted 500 times with LB medium. Viability was evaluated using propidium iodide staining and bacteria DNA were labeled with Syto9 (Invitrogen, ex./em.: 485nm / 598 nm). Cells were incubated in LB medium 30 min at 37°C with propidium iodide (PI) (15 μg/ml) and Syto9 (5 μM), then washed and resuspended in PBS. Propidium iodide control cells were treated with 1 mg/mL PmB during staining. Proportion of PI positive events were appreciated through Syto9 positive population (n ≥ 10 000 events) within SSC/FSC selected bacterial population. Analyses were performed with FACS Calibur flow cytometry (Becton Dickinson).

### Dilution spot assay

Samples were taken from 24 hours final cultures and serial dilutions were performed in PBS. Ten microliters of diluted cultures were spotted on LB agar plate and incubated overnight at 37°C. Experiments were done in technical triplicates within biological triplicates.

### Western blot analysis

Bacteria growth was performed for 24h in LB medium with or without 25 μg/mL PmB. Pellets and supernatant from 1 mL culture were separated by centrifugation at 5500 x g. One mL of supernatant or 1 mL of total culture (bacteria and supernatant compartment not divided) extracts were precipitated at 4°C with trichloroacetic acid (TCA) at 87.5 μg/mL, then centrifuged 20 min at 20,000 x g and suspended in 50 μL of phosphate saline buffer. The pellet from 1 mL culture was suspended in 100 μL of phosphate saline buffer. Then, the proteins were subjected to polyacrylamide gel electrophoresis (10 μL of pellet or 5μL of supernatant or total culture extract) using the method described by Laemmli (1970) before blotting onto a polyvinylidene difluoride (PVDF) membrane. Proteins were identified using anti-flagellin (αFla) and anti-cAMP receptor protein (αCRP) polyclonal antisera [42, 43]. Anti-rabbit horseradish peroxidase-conjugated antibody preparation (Invitrogen) was used as a secondary antiserum at a final dilution of 1:20,000. The ECL Prime chemiluminescence system (GE Healthcare) was used to detect chemiluminescence, which was recorded using an Amersham imager 600 (GE Healthcare). Quantification was performed using the signal intensity with ImageJ software and expressed as the percentage of the signal intensity relative to the control without PmB.

### Proteomic analysis

*V*. *cholerae* A1552 was grown with or without of 12 μg/mL of PmB in LB supplemented with 2% NaCl. When the bacterial culture reached an OD_600nm_ of 2, the cultures were centrifuged at 5500 x g. The supernatants (Secretome) were precipitated at 4°C with 87.5 μg/mL of TCA. Bacterial cell pellets and precipitated supernatants were reconstituted in 50 mM ammonium bicarbonate with 10 mM TCEP [Tris(2-carboxyethyl)phosphine hydrochloride; Thermo Fisher Scientific], and vortexed for 1 h at 37°C. Chloroacetamide (Sigma-Aldrich) was added for alkylation to a final concentration of 55 mM. Samples were vortexed for another hour at 37°C. One microgram of trypsin was added, and digestion was performed for 8 h at 37°C. Samples were dried down and solubilized in 5% ACN-0.2% formic acid (FA). The samples were loaded on a 1.5ul C18 precolumn from Optimize Technologies connected directly to the switching valve. They were separated on a home-made reversed-phase column (150-μm i.d. by 150 mm) with a 56-min gradient from 10 to 30% ACN-0.2% FA and a 600-nl/min flow rate on a Ultimate 3000 (Eksigent, Dublin, CA) connected to an Q-Exactive Plus (Thermo Fisher Scientific, San Jose, CA). Each full MS spectrum acquired at a resolution of 70,000 was followed by 12 tandem-MS (MS-MS) spectra on the most abundant multiply charged precursor ions. Tandem-MS experiments were performed using collision-induced dissociation (HCD) at a collision energy of 27%. The data were processed using PEAKS 8.5 (Bioinformatics Solutions, Waterloo, ON) and a vibrio cholerae database (96965 entries). Mass tolerances on precursor and fragment ions were 10 ppm and 0.01 Da, respectively. Variable selected posttranslational modifications were carbamidomethyl (C), oxidation (M), deamidation (NQ) and acetyl (N-ter). The data were visualized with Scaffold 4.3.0 (protein threshold, 99%, with at least 2 peptides identified and a false-discovery rate [FDR] of 1% for peptides).

### RT-qPCR analysis

RNA was extracted in early log phase from A1552 and MO10 grown with or without 25 μg/mL PmB and A1552 Δ*rpoN* as negative control for flagellin expression (Qiagen RNAprotect bacteria reagent and RNeasy Protect Bacteria kits and Thermofisher TURBO DNA-free kit). RNAs were retrotranscribed to cDNA (Qiagen QuantiNova Reverse Transcription kit). Flagellin genes *flaA* (primers: *flaA*-F2: 5’-CCGTTTGACCGTTGATGTA-3’ and *flaA*-R2: 5’-CTCGTGACCTGAAGTTTGAA-3’) and *flaE* (primers: flaE-F3 5’-TCTGCGTCCGTGAATGAAAA-3’, *flaE*-R3 5’-CTAAACTGCCACTGAACGACAA-3’) and *mshA* (primers: *mshA*-F3 5’- CCTGGAACAGTTATTGATGGC-3’, *mshA*-R3 5’- ACTCACTCGAAGTATCTAGCG-3’) expressions were evaluated in RT-qPCR with SYBR green (Qiagen QuantiNova SYBR Green PCR Kit and measured on Corbett RotorGene 6000) and *recA* was used as house housekeeping gene using the primers form Wardman et al. [44]. RT-qPCR mix contains 10 μl 2x SYBR Green mix, 2 μl template, 2.8 μl of 5 μM forward and reverse primers and 5.2 μl H_2_O for a final volume of 20 μl. RT-qPCR reactions were performed as following: 1x 95°C for 10 min; 40x 95°C 30 sec, 58°C 8 sec, 72°C 20 sec. Then melting curves were performed 72°C for 90 sec, and 72°C to 95°C at 1°C per step and 5 sec per step. Each sample was analyzed in technical triplicates and biological duplicates. No template controls were also added for each gene analyzed in each run. A1552, MO10 and A1552 Δ*rpoN* ΔCt were calculated for *flaA* and *flaE* ΔCt were calculated as: ΔΔCt = [*Ct*_*flaA* or *flaE*_−*Ct*_*recA*_]_+PmB_—[*Ct*_*flaA* or *flaE*_−*Ct*_*recA*_]_-PmB_. Standard deviation was calculated on each ΔΔCt combinations between technical replicates.

### Colony forming unit

A1552 and MO10 strains were grown with or without PmB, samples were taken from 2 hours final cultures and serial dilutions were performed in PBS. One hundred microliters of diluted cultures (10^−5^, 10^−6^, 10^−8^ and 10^−8^) were plated on LB agar medium and incubated overnight at 37°C. Experiments were done in technical triplicate for each dilution and biological duplicate.

## Results

### Sub-inhibitory concentration of polymyxin B inhibits *V*. *cholerae* biofilm formation at the initial attachment stage

**Fig 1:**
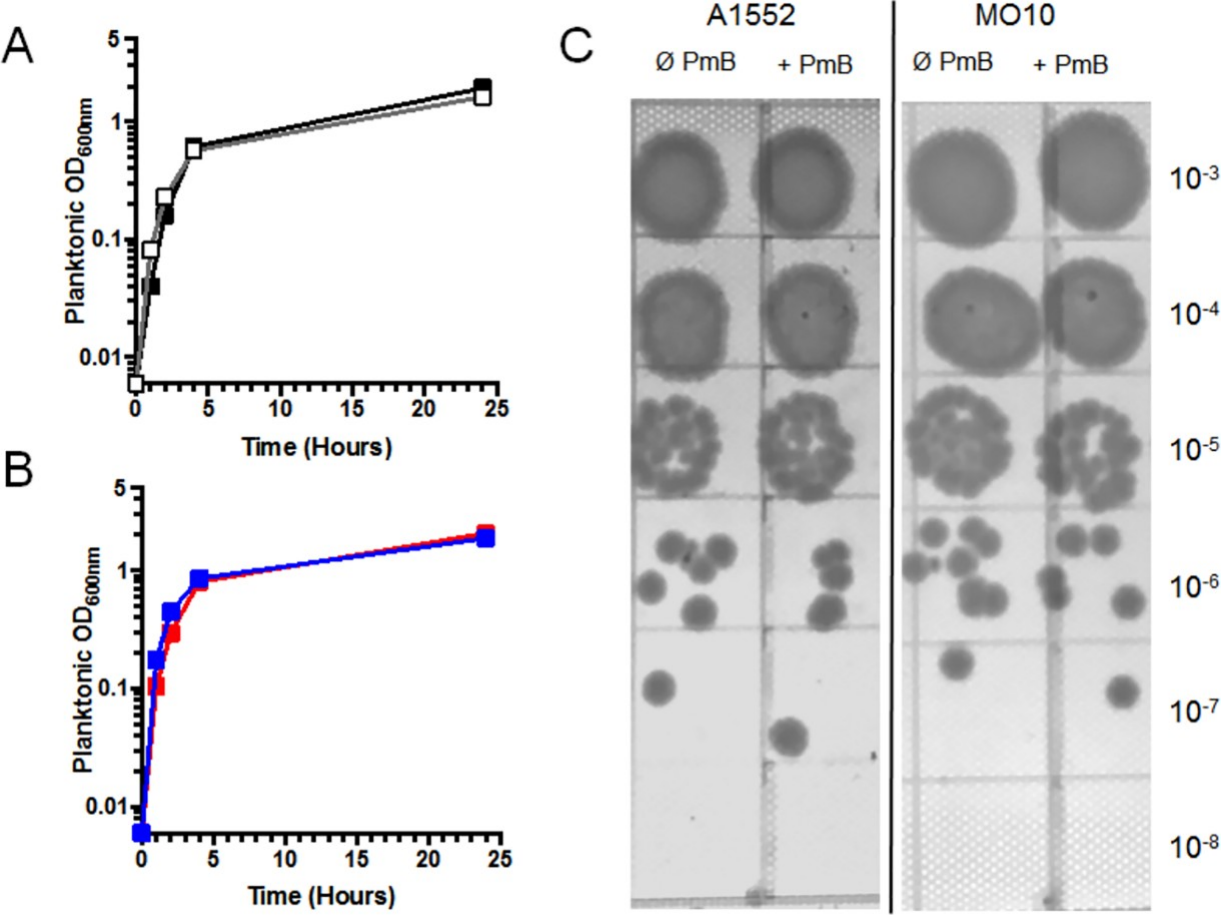
A1552 and MO10 growth are not impaired by 25 μg/mL of polymyxin B. (A) A1552 and MO10 growth curves, means of technical duplicates within biological triplicates. A1552 grown without PmB (white) and with 25 μg/mL PmB (black); (B) MO10 grown without PmB (blue) and with 25 μg/mL PmB (red). (C) A1552 and MO10 dilution spot assay with or without 25 μg/mL PmB. Representative picture of experiments done in technical triplicates within biological triplicates.

**Fig 2:**
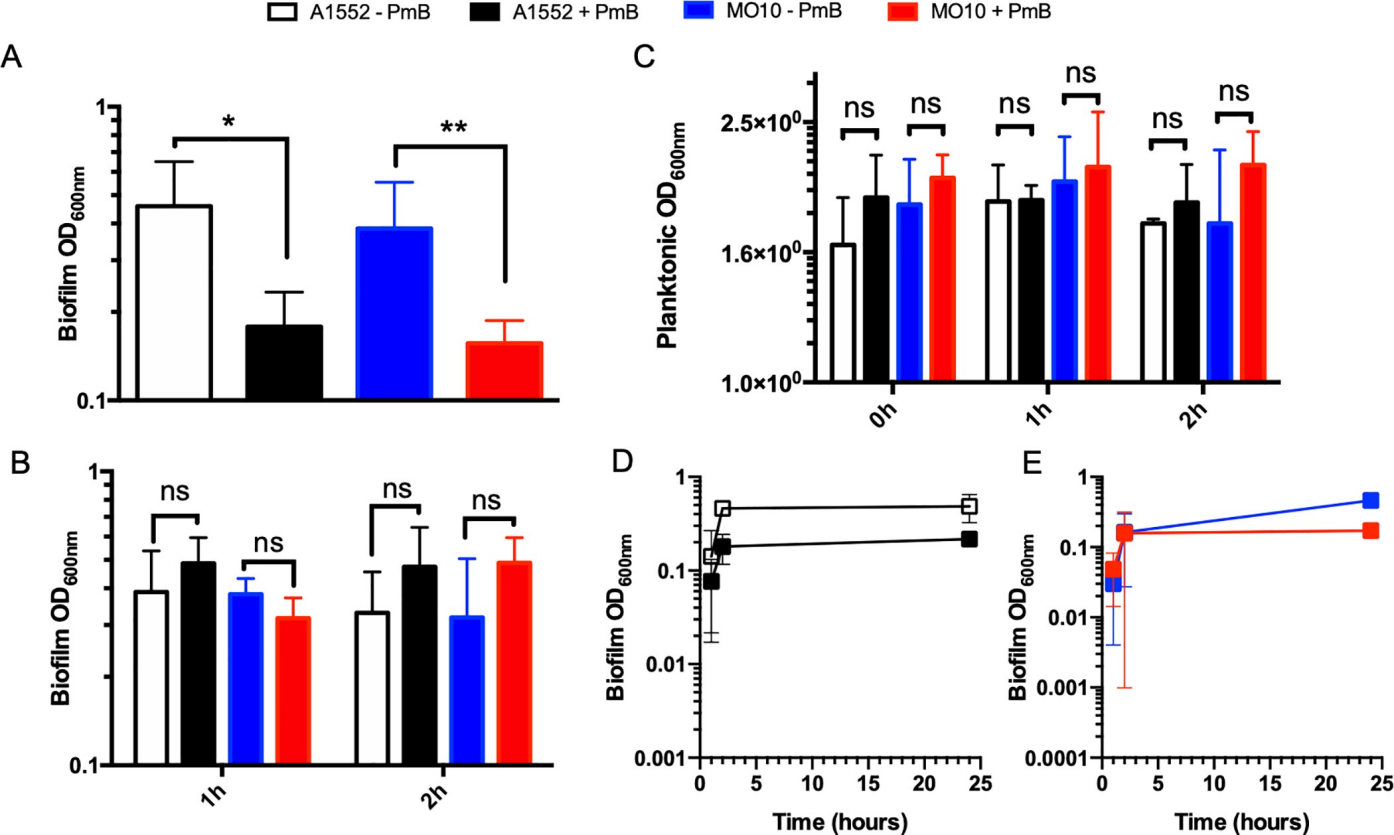
Sub-inhibitory polymyxin B concentration inhibits *V*. *cholerae* A1552 and O139 biofilm formation at the early adhesion stage. (A) and (B) Biofilm quantification and (C) planktonic optical density of A1552 and MO10 strain cultures after 24h incubation. (A) Polymyxin B (PmB) or water (control) were added from the beginning, and (B) at one hour (1h) or two hours (2h) after culture starts. A1552 without (-) PmB (white) and A1552 with (+) 25 μg/mL PmB (black); MO10 without (-) PmB (blue) and MO10 with (+) 25 μg/mL PmB (red). Biofilm formation kinetics of A1552 (C) and MO10 (D), PmB is added at the beginning of culture. (A, B, C and D) All results were done in technical duplicates within biological triplicates. Data correspond to mean ± SD. (A and B) Two-Way ANOVA with Tukey correction was performed to compare each condition (ns; * P < 0.01; ** P < 0.005).

To determine the effect of sub-inhibitory concentration of PmB on biofilm formation by *V*. *cholerae*, we quantified the biofilm of two strains belonging to the O1 and O139 serougroups, *i*.*e*. A1552 and MO10, respectively. To do so, the bacteria were grown in presence of 25μg/mL of PmB (¼ of the MIC), a concentration that did not alter the growth rate or yield (Fig 1A and 1B). A dilution spot assay on mid-exponential and stationary phase cultures was performed and the results demonstrate that no difference in CFU can be observed in presence or in absence of PmB (Fig 1C and S4 Fig). Then, a quantification of the biomass on a 24h biofilm by crystal violet has been performed in presence and in absence of sub-inhibitory concentration of PmB. As demonstrated in Fig 2A, the quantity of biofilm is significantly reduced in presence of PmB, with a decrease of around 60% for both strains. The turbidity in the medium surrounding the biofilm has also been measured to estimate the quantity of planktonic bacteria. The results show that the turbidity is not modified in presence of PmB (Fig 2C).

To identify at which stage of biofilm formation the inhibition occurs, we analyzed the effect of PmB addition on a preformed biofilm. At this stage, the initial bacterial adhesion stage is complete, and the maturation stage leading to the development of a mature biofilm is initiated. To do so, we first determined the kinetic of the biofilm formation for both *V*. *cholerae* strains. Our results show that after 1h, the biofilm has started to form and is quantifiable using the crystal violet method, meaning that the initial stage of biofilm formation is achieved (Fig 2D and 2E). Then, we analyzed the effect of an addition of PmB at sub-inhibitory concentration (25 μg/mL) on the 1h-preformed biofilm. The results demonstrate that the biofilm formation is not affected when the PmB is added on a preformed biofilm (Fig 2B). Therefore, our results demonstrate that the presence of PmB at sub-inhibitory concentration results in a reduction of the biofilm formation at the initial stage, suggesting an impairment of the bacterial adhesion.

### Reduction of motility in presence of polymyxin B

The flagella are essential for the initiation of biofilm formation in *V*. *cholerae* by providing the movement leading to the contact of the bacterial cell with the surface, by physically adhering to the surface and by signaling the bacterial adhesion, leading to the secretion of the biofilm matrix components. Therefore, since flagella are essential effectors of the initial adhesion steps of biofilm formation, we analyzed the effect of sub-inhibitory concentration of PmB on flagellation.

**Fig 3:**
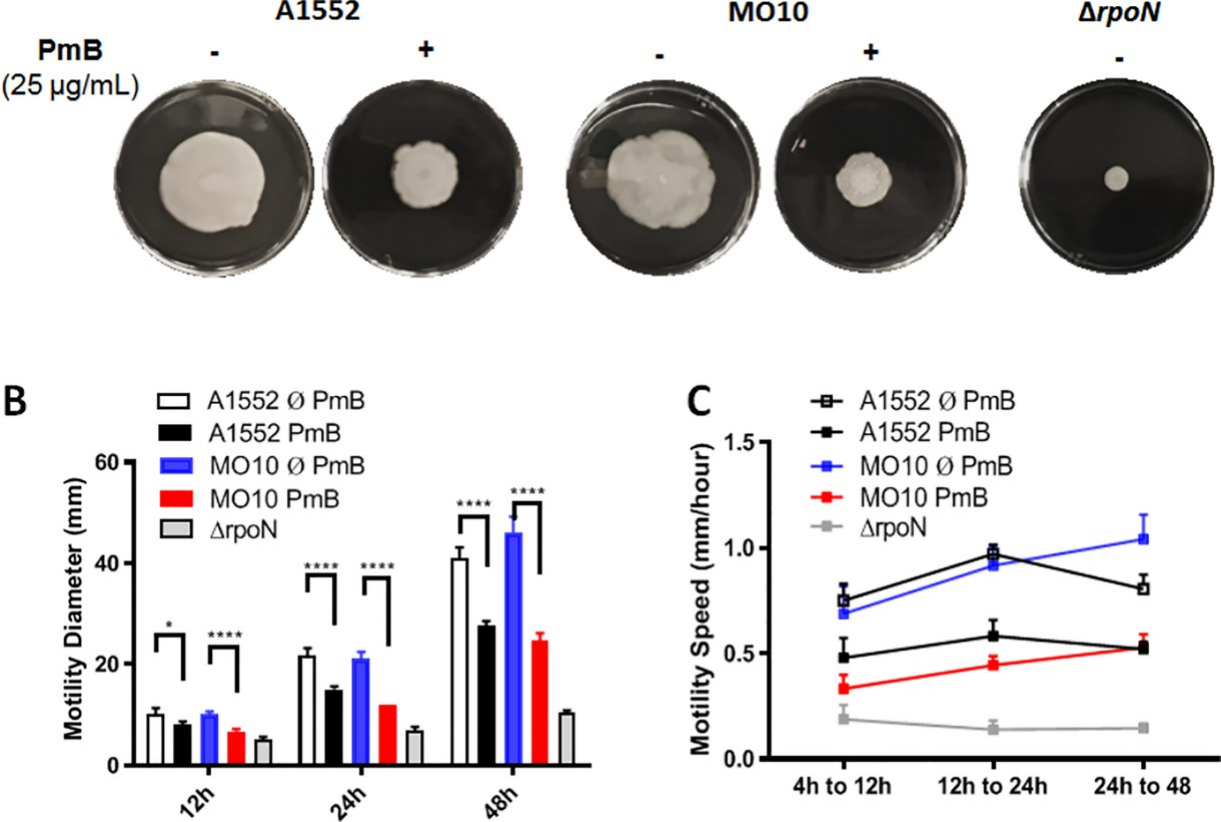
Polymyxin B inhibits *V*. *cholerae* motility. (A) Swimming motility assays. Aliquots of A1552 or MO10 in exponential phase of growth were spotted in the center of a Petri dish containing LB LB agar 0.3%. An A1552 Δ*rpoN* was used as non-motile control. Photographs were taken after 48h incubation at 37°C. (B) A1552 and MO10 *V*. *cholerae* strains swimming diameter on plates at 12h, 24h and 48h. A1552 without (white) and with (black) 25 μg/mL polymyxin (PmB); MO10 without (blue) and with (red) 25 μg/mL PmB. (C) Speed motility in mm/hour calculated based on the growth diameter difference on Petri dishes containing LB agar 0.3%. Data correspond to technical triplicates within biological triplicates mean ± SD. Two-Way ANOVA with Tukey correction was performed to compare each condition (**** P < 0.0001).

Because of the essential role of flagella in motility, a determination of the effect of sub-inhibitory concentration of PmB on *V*. *cholerae* A1552 and MO10 motility was performed. To do so, the *V*. *cholerae* strains A1552 and MO10 were grown to an early exponential phase in presence of PmB and 3 μL were spotted on soft agar containing 25 μg/mL (sub-inhibitory concentration) of PmB and incubated at 37°C. A control without PmB has been performed in parallel. The motility was determined after 12h, 24h and 48h. Our results demonstrate a statistically significant reduction of the bacterial motility at 12, 24 and 48h for both strains upon PmB supplementation, with a final diameter after 48h reduced from an average of 33% and 46% for *V*. *cholerae* O1 and O139, respectively (Fig 3A and 3B). Using the mutant of the positive regulator of flagellin expression RpoN (Δr*poN)* as non-motile control [45], we determined that the motility is not entirely abolished in presence of PmB. Calculation of the motility speed at different time points (Fig 3C) demonstrated that *V*. *cholerae* A1552 reaches its fastest motility between 12 and 24h with a tendency to reduce the speed between 24 and 48h. Conversely, MO10 strain motility is constantly accelerating all over the 48h of the experiment time course. The trend of the bacterial speed is not altered in presence of PmB, but the motility speed is reduced at all the different time points tested (12h, 24h, 48h), suggesting a constant effect of PmB on *V*. *cholerae* motility (Fig 3C). In addition, a dose-dependent effect of the PmB motility has been observed with a reduction for concentration as low as 3.12 μg/mL at 24h (S1 Fig). Similar results have been obtained using colistin, another antimicrobial peptide, on *V*. *cholerae* A1552 motility (S1 Fig). Thus, growing *V*. *cholerae* O1 and O139 strains in presence of sub-inhibitory concentration of PmB results in a significant dose-dependent reduction of the motility.

### Sub-inhibitory concentration of polymyxin B does not interfere with the flagellins expression

**Fig 4:**
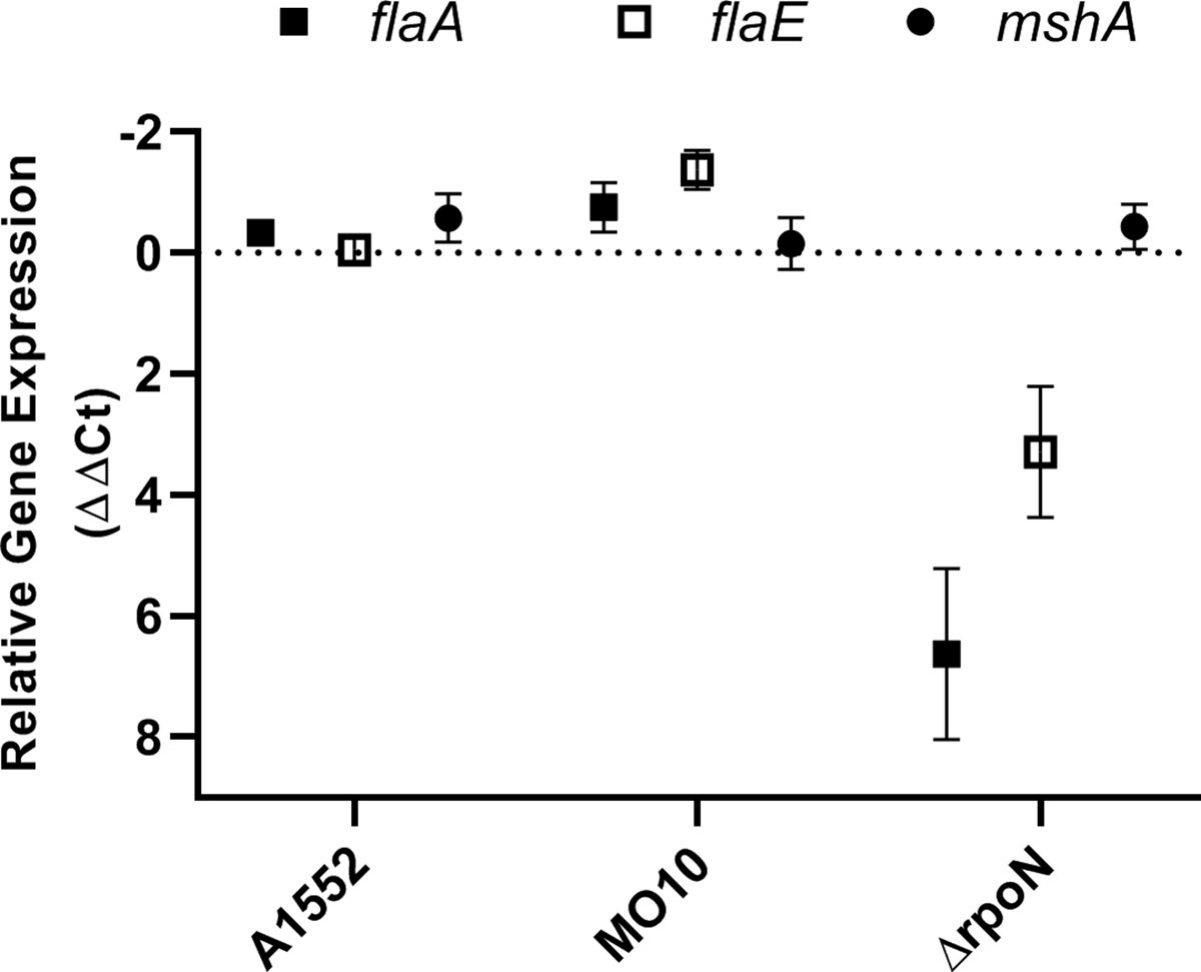
Sub-inhibitory concentration of polymyxin B does not reduce the expression of the flagellin and the MSHA pili genes. Relative gene expression (ΔΔCt) for *flaA*, *flaE* and *mshA* in A1552 and MO10 in presence of PmB 25μg/mL in exponential phase of growth in comparison with a control without PmB. The Δ*rpoN* strain was used as negative control of the flagellins expression. Data correspond to technical triplicates within biological duplicates mean ± SD. A positive ΔΔCt value indicates an increase in gene expression, conversely a negative ΔΔCt value indicates a decrease in gene expression.

**Fig 5:**
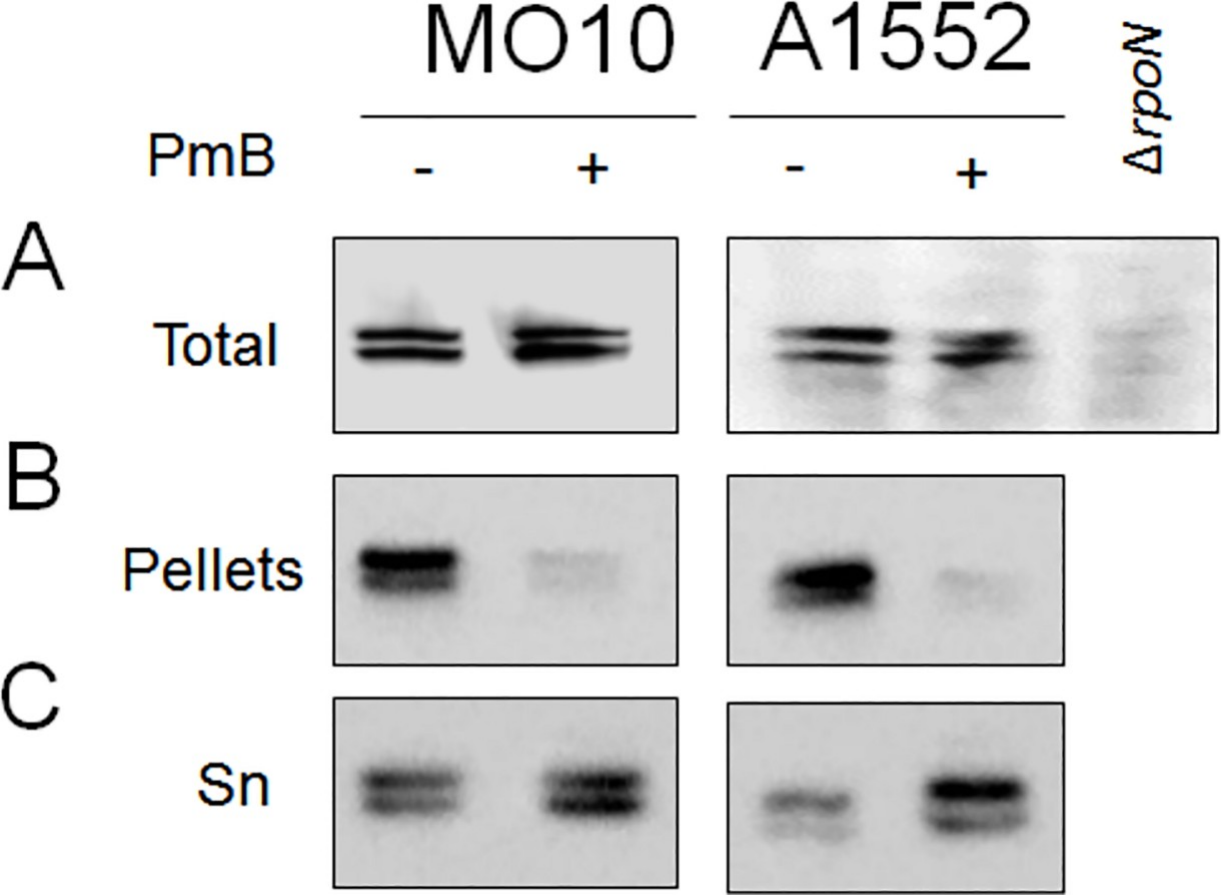
Modification of the flagellin location in presence of sub-inhibitory polymyxin B. Presence of flagellin was evaluated by western-bot using anti-flagellin antibody on A1552 and MO10 in total culture (bacteria and supernatant compartment not divided) (A), in cell pellets (B) and in culture supernatant (C) (Sn), in absence (-) and presence (+) of 25 μg/mL PmB. A negative control of flagellin signal consisting of the Δ*rpoN* mutant of A1552 is shown for the total culture extract. Picture is representative of ≥ three biological replicates.

Since we observed a reduction of the motility of *V*. *cholerae* in presence of sub-inhibitory concentration of PmB, we examined whether this reduction was due to a global down regulation of flagella-related genes expression. Thus, real-time PCR analyses were performed in presence of sub-inhibitory concentration of PmB and compared with the control without PmB. The housekeeping gene *recA* was used as internal control. The main component of the flagella are the flagellin subunits and, in *V*. *cholerae*, they are organized in two distinct operons, *i*.*e*., *flaAC* and *flaEDB*. Therefore, we determined the relative expression of *flaA* and *flaE*, the first genes of both operons as markers of the global flagellin expression. Our results demonstrated that the expression of *flaA* and *flaE* is not reduced in presence of sub-inhibitory of PmB for both strains (Fig 4). Conversely, an increase of *flaE* expression is observed in MO10 in presence of sub-inhibitory concentration of PmB (Fig 4). In addition, we also measured the expression of *mshA* as a marker of the expression of the MSHA pili, the other structural element involved in V. cholerae adhesion and biofilm formation. We observed no significant effect of the PmB supplementation on *mshA* expression (Fig 4). As expected, the Δ*rpoN* control display a significant down regulation of *flaA* and *flaE*. To confirm the real-time PCR results, we quantified the flagellin protein in *V*. *cholerae* A1552 and MO10 total culture extracts by western-blot. Our results demonstrated that no difference in flagellin quantity can be observed in the total culture extract (Fig 5A). Altogether, these results suggest that sub-inhibitory concentration of PmB does not reduce the expression of the flagellin genes or the quantity of flagellin produced.

### The quantity of flagellin associated with *V*. *cholerae* cells is reduced in presence of polymyxin B

To understand the motility reduction, we analyzed the flagellin localization in the total culture extract. To do so, we separated the bacterial cells from the secreted proteins by centrifugation and determined the relative abundance of the flagellin by western-blot in both compartments. Our western-blot results show a decrease of 86% and 83% of the signal corresponding to the flagellins upon PmB supplementation in *V*. *cholerae* A1552 and MO10 cells, respectively. Conversely, an increase of the flagellin in the supernatant upon PmB supplementation is observed in the western-blot analysis of secreted flagellin, especially in the *V*. *cholerae* O1 strain A1552 (Fig 5A–5C). The signal quantification demonstrated an increase of 156% and 45% of the signal for A1552 and MO10, respectively. Using a CRP antibody, we confirmed that the supernatant preparation is free of cytoplasmic proteins (S2 Fig). A quantitative mass spectrometry analysis of the secretome (Table 1) confirmed that FlaA, FlaB, FlaC and FlaD but not FlaE were detected in the secretome of *V*. *cholerae* and their abundance increases between 1.4 and 2.1 in presence of PmB in comparison with the control without PmB (Table 1).

**Table 1:** Polymyxin B increase flagella components in A1552 secretome^a^.

Protein	Relative abundance^b^
FlaA	2.1
FlaB	1.6
FlaC	1.8
FlaD	1.4
FlaE	*N*.*D*.

^a^ Quantitative mass-spectrometry analysis in presence or absence of PmB. Relative abundance of the five flagellin subunits in the culture supernatant of *V*. *cholerae* O1 strain A1552 grown in presence of 12,5 μg/mL of PmB in comparison with the control without PmB. *(N*.*D*.: *not detected)*.

^b^ Fold change difference in terms of spectrum counts between A1552 grown with 12,5 μg/mL PmB and the control grown without PmB.

**Fig 6:**
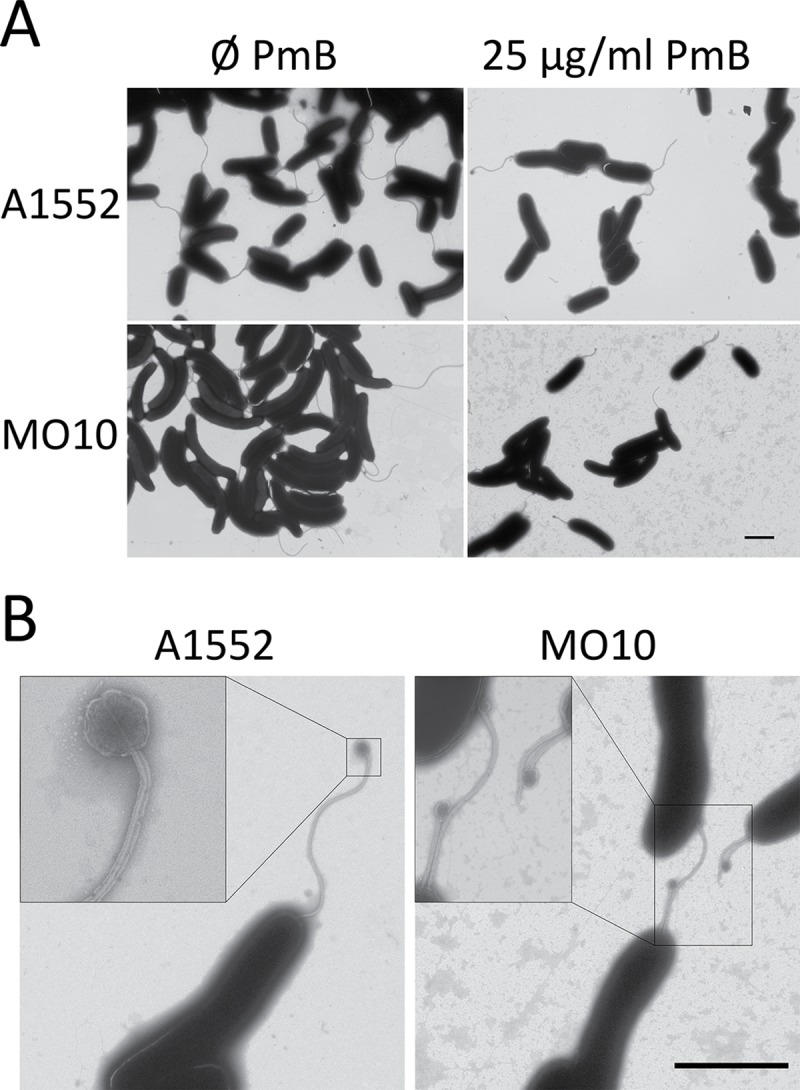
Polymyxin B sub-lethal concentration causes A1552 and MO10 flagella loss and misshaping. (A) Transmitted electronic microscopy pictures of *V*. *cholerae* A1552 and MO10 strain, grown in absence (-) or in presence (+) of 25 μg/mL PmB. Pictures are representative of 3 replicates. (B) Transmitted electronic microscopy pictures of *V*. *cholerae* A1552 and MO10 strains, grown in presence 25 μg/mL PmB. Scale bar corresponds to 2 μm. Pictures show elements seen in 3 biological replicates.

Finally, electronic microscopy observations of *V*. *cholerae* O1 and O139 strains in early exponential phase of growth were performed. Images indicate that a supplementation in PmB during bacterial growth is associated with a reduction of the number of flagellated bacterial cells, especially for A1552 with a reduction of 57% of the number of flagellated bacteria (Fig 6A, and S1 Table). Regarding MO10, the number of flagellated cells is not drastically reduced (S1 Table). However, the flagella observed in presence of PmB are significantly shorter than the control without PmB (Fig 6A). Finally, a bulb-like structure can be observed at the tip of some of the flagella attached to bacterial cells, especially when they are grown with PmB, with an increase of around 2395% and 355% for A1552 and MO10 respectively. A magnification of this bulb structure is shown in Fig 6B.

Altogether, our results strongly suggest that a large portion of the flagellin subunits are localized in the secretome instead of attached to the bacterial cells upon PmB supplementation, which can be explained by a reduction of the number of flagellated cells or by an increase in the proportion of misshaped flagella.

### Addition of sub-inhibitory concentration of AMP does not induce major membrane damages

Since we observed an increase of the flagellin quantity in the supernatant fraction of the *V*. *cholerae* O1 culture in presence of sub-inhibitory concentration of PmB, we wondered if the presence of PmB may have affected the cell wall integrity even though the bacterial growth was not affected. To do so, we performed flow cytometry analysis using propidium iodide, a marker of the cell wall integrity. Three independent experiments were performed on mid-exponential phase culture for both *V*. *cholerae* O1 and O139 strains grown in presence and in absence of sub-inhibitory concentration of PmB (25 μg/mL). For both strains, no significant increase of the propidium iodide positive bacteria was observed (Table 2 and S3 Fig). The PI^+^ population represents 3.4% ± 2.0% and 3.9% ± 0.7% without PmB, and 4.0% ± 2.2% and 2.0% ± 1.0% with 25 μg/mL PmB for *V*. *cholerae* A1552 and MO10, respectively. A positive control consisting of a short treatment with lethal concentration of PmB has also been performed and demonstrated that, as expected, the PmB can form pores inducing major damages in *V*. *cholerae* cell wall, with 87.8% and 91.7% of propidium iodide positive cells for *V*. *cholerae* A1552 and MO10, respectively (Table 2). Therefore, our results clearly establish that growing *V*. *cholerae* in presence of sub-inhibitory concentration of PmB did not drastically alter the cell wall integrity.

**Table 2:** A1552 and MO10 membrane integrity is not impaired by 25 μg/mL polymyxin B^a^.

		N. SYTOX9 pos.Events	Freq. of SSC/FSC sub. (%)	Freq. of SYTO9 pos. (%)	Geometric Means	Freq. of PI pos. (%)
**A1552 Ø PmB**	Mean	29317,67	20,67	40,27	6,11	3,36
	SD	12663,00	6,45	3,88	2,87	2,02
	N	3,00	3,00	3,00	3,00	3,00
**A1552 + PmB**	Mean	20679,33	32,20	39,87	8,24	3,99
	SD	10817,08	6,98	6,46	1,87	2,15
	N	3,00	3,00	3,00	3,00	3,00
**MO10 Ø PmB**	Mean	28227,33	17,30	78,83	-4,10	3,86
	SD	963,44	3,44	5,28	0,81	0,67
	N	3,00	3,00	3,00	3,00	3,00
**MO10 + PmB**	Mean	27037,00	24,23	76,00	-8,83	2,01
	SD	2938,94	5,76	10,79	3,87	1,00
	N	3,00	3,00	3,00	3,00	3,00
**A1552 Pos. Control**	Mean	48687,00	28,70	74,70	361,00	87,80
	SD	–	–	–	–	–
	N	1	1	1	1	1
**MO10 Pos. Control**	Mean	45628,00	25,20	72,30	464,00	91,70
	SD	–	–	–	–	–
	N	1	1	1	1	1

^a^Membrane viability of A1552 and MO10 *V*. *cholerae* strains grown with and without PmB were appreciated via flow cytometry experiments using propidium iodide (PI) and DAPI probes. PI positive events represent cells with impaired membrane integrity. Cell populations were primarily gated in SSC/FSC then DAPI positive strains were selected. Frequency in parental population (≥ 35000 events), geometric mean (geomean) and proportion of PI positive and negative cells were measured in biological triplicates ± SD. Two-Way ANOVA with Tukey correction was performed to compare geomean and PI positive and negative cell proportion of the two strains grown with or without PmB. Only comparison of PI signal geomean between A1552 grown with (+) and without PmB (-) show a significant difference with (P < 0.0001).

## Discussion

Flagellum is the apparatus responsible for the movement of the bacteria, and the direction is guided by the chemotaxis system. In several pathogens, including *V*. *cholerae*, flagella are important for the colonization and the virulence inside the host [18, 46–48]. In the context of antibiotic resistance, a global effort is currently ongoing to discover alternatives to antibiotics including new antimicrobial drugs, phage therapy or virulence blockers [49]. Several studies have focused on the antimicrobial peptides for their potential role as an alternative to antibiotics [50–52]. An effect of antimicrobials on bacterial motility has been reported for several Gram-negative bacteria. In *Campylobacter jejuni*, the carvacrol, a component of essential oil with antibacterial activity, at sub-inhibitory concentration inhibits bacterial motility and invasion of eukaryotic cells without affecting the flagella structure [53]. In *Pseudomonas aeruginosa* and *Burkholderia cenocepacia*, the motility reduction in presence of cationic antimicrobial peptides was associated with a down-regulation of the flagella synthesis [54, 55]. Similarly, the antibiotic azythromycin reduces the flagellation of *Salmonella enterica* [56]. Here, we observe a diminution of the motility of *V*. *cholerae* in presence of sub-inhibitory concentration of PmB. Even though the motility of *V*. *cholerae* is not entirely inhibited in comparison with the aflagellated mutant Δ*rpoN*, it remains impaired at concentration as low as 3.12 μg/mL, which represents 1/16 of the minimal inhibitory concentration in the conditions tested here. In addition, we observed a similar dose-dependent effect with colistin, another AMP, which suggests that this effect is not limited to PmB.

*V*. *cholerae* has a single polar flagellum essentially composed of the flagellin subunits and coated by a membrane sheath [19, 21]. Here, we observed a decrease in the quantity of flagellin associated with the bacterial cells. This decrease could be due to a reduction in the number of flagellated bacteria or a reduction of the flagella size. Our electronic microscopy analysis suggests that the proportion of aflagellated bacteria increases and that the flagella length decreases upon PmB supplementation. The reduction of the flagellin associated with the bacterial cells is correlated with an increased abundance in the secretome, as demonstrated by immunoblot and mass-spectrometry analysis. Indeed, in the mass-spectrometry analysis, 4 out of 5 flagellin subunits, *i*.*e*. FlaA, FlaB, FlaC and FlaD, are more abundant in presence of PmB in comparison with the control without antimicrobial peptide. FlaA is the only flagellin subunit essential for the flagellum synthesis in *V*. *cholerae* [19, 20]. In addition, we observe no down-regulation of the flagellin of the MSHA pili expression and no modification of the global flagellin quantity in the whole culture extracts. However, the increase in flagellin in the secretome upon PmB supplementation is equivalent to the amount lost in the cell extract sample, according to the immunoblot quantification analysis. Therefore, the reduction of the number of flagellated cells is not due to a global down regulation of the flagella synthesis, but more probably to a flagellum miss assembly or miss-anchoring.

The sensitivity of the *Vibrio* to antimicrobial peptides varies according to the strain. Regarding the pathogenic *V*. *cholerae*, the strains belonging to the O1 serogroup are divided in two biotypes that display different sensitivity toward the PmB. Indeed, the *V*. *cholerae* O1 Classical strains are sensitive to PmB whereas the El Tor strains are resistant, a phenotype that has been historically used to differentiate between the biotypes [57]. The O139 serogroup is also resistant to PmB. In addition, it has been demonstrated that *V*. *cholerae* O1 El Tor and Classical use different pathways to control biofilm formation [58]. In the condition of biofilm formation used here, we were unable to observe biofilm formation with the Classical biotype strains tested (569B and O395) after 72 hours. Therefore, we focused on the O1 El Tor A1552 and the O139 MO10 strains, which are both resistant to PmB and form strong biofilm after 24h in the conditions used in this study.

Resistance to antimicrobial peptides in *Vibrio* species involves different mechanisms, some of them most probably occurring simultaneously. Those mechanisms are membrane remodeling, AMP trapping by the bacterial membrane vesicles, induction of an envelope stress response, efflux systems and inhibition of antimicrobial peptide expression [59]. Conformational modification of the outer membrane leading to the OmpU-dependent activation of the alternative sigma factor σE has also been reported in presence of antimicrobial peptides [60]. The σE regulon of *V*. *cholerae* is involved in antimicrobial peptide resistance, intestinal survival and virulence [60, 61]. In addition, we have previously reported a modification of the outer membrane vesicles with the stronger association of the biofilm matrix protein Bap1 to the OmpT porin at the surface of the vesicles, which is responsible for the cross-resistance to the human cathelicidin LL-37. Altogether those studies clearly indicate that the membranes of *V*. *cholerae* undergo structural and protein abundance modifications under antimicrobial pressure. In this study we demonstrate that *V*. *cholerae* flagellin subunits, the main component of the flagella, are less associated with the bacterial cells. The flow cytometry analysis established that *V*. *cholerae* cell wall is not damaged in presence of sub-inhibitory concentration of PmB. However, it is possible that the PmB-dependent membrane remodeling might result in a miss-anchoring of the flagella to the bacterial cell wall, leading to the release of the flagella in the supernatant and decrease of bacterial motility.

The PmB is a cationic AMP that binds to the lipid A of the lipopolysaccharide of the Gram-negative bacteria [62]. At high concentration, it was demonstrated that PmB can induce a membrane depolarization leading to the formation of pores in the cytoplasmic membrane [63]. As discussed above, no major membrane damages have been observed in this study upon PmB supplementation. However, at lower concentration of PmB, an accumulation of lipophilic anions in the cytoplasmic membrane coupled with a leakage of the potassium anion has been reported in *E. coli [63]*. This leakage is responsible for the dissipation of the potassium gradient on the cytoplasmic membrane without formation of pores. Similarly to *E*. *coli*, a disturbance in the ion flux in the cytoplasmic membrane might also occur in presence of sub-inhibitory concentration of PmB in *V*. *cholerae*. This membrane potential disturbance could lead to a compromised ability to assemble the flagellum and to the free secretion of the flagellin instead of their assembly to form the flagellum. Therefore, the increase of flagellin abundance in the secretome in correlation with a decrease in association with *the V*. *cholerae* cells might also be due to a compromised ability to assemble the flagellum leading to the secretion of free flagellin subunits.

Besides the reduction of the number of flagellated cells, we also observed a large proportion of flagella with bulb-like structures upon PmB supplementation, but very rarely in the cultures without the antimicrobial peptide. Basal bulbs have already been observed in *V*. *metscnikovii* flagella, which consist of a single spherical fragment of the membrane [64]. In *V*. *fisheri*, bead-like structures have been observed at the distal end of the flagellum [65], but their structure seems to be very different from what we observed in this study. Bulb-like structures on the flagella are, however, characteristic to *Helicobacter pylori*, where it is a common structure under normal growth conditions [66]. The flagella of *H*. *pylori* is important for motility and has a role in colonization of the mucosa [67]. Similarly to *V*. *cholerae*, the flagella of *H*. *pylori* are coated with a sheath composed of LPS and proteins [21, 68]. A role for this sheath in the release of immunogenic LPS has been proposed in *V*. *fisheri* [69]. However, the role of the bulb-like structure, which seems to be a dilatation of the flagella sheath at the tip of the flagella of *H*. *pylori* [66], and the impact of this structure on the motility of the bacteria, remains unknown.

Biofilm formation is a common virulence trait of bacterial pathogens [70]. In *V*. *cholerae*, biofilm formation is initiated by the movement of the flagellum, which allows the bacteria to reach the support and initiate the first reversible attachment. Upon contact, *V*. *cholerae* O1 flagellum induces a counter-rotation leading to the contact of the mannose-sensitive hemagglutinin pili with the surface. At this stage, the attachment is irreversible leading to the initiation of the early development stage of biofilm formation [17]. This initiation is mediated by the flagellum rotor, which induces the expression of the exopolysaccharide, the main component of the biofilm matrix [71]. In this study, we have clearly established that the inhibition of the biofilm formation by sub-lethal concentration of polymyxin B is occurring at the initial stage of the biofilm formation, as demonstrated by the experiment on preformed biofilm. We further investigated the effect of the PmB on the flagellum and demonstrated that the number of aflagellated cells increases together with an augmentation of the flagellin in the secretome. These observations are correlated with a significant reduction of motility. In addition, no modification in the flagellin genes expression was observed. Therefore, our results strongly suggest that, in presence of sub-inhibitory concentration of PmB, the reduction in biofilm formation is most likely due to an increase in the proportion of aflagellatted or misshaped flagella for *V*. *cholerae* A1552 cells, and shorter and/or misshaped flagella for MO10 strain. These phenotypes most probably explain the impaired ability to attach to a surface and to form biofilms for both strains.

In the context of antibiotic resistance, inhibiting biofilm formation has been proposed as a new way to cope with multidrug-resistant pathogenic bacteria [72–75]. An inhibition of the biofilm formation by synthetic cationic antimicrobial peptides has already been reported for the *Pseudomonas aeruginosa*, *Burkholderia cenocepacia*, and *Listeria monocytogenes* [54]. Similarly, a reduction in biofilm formation has been observed in *Pseudomonas aeruginosa* in presence of PmB but the mechanism of biofilm inhibition is still unknown. This reduction is accentuated by the combination of PmB with Gramicin S, another AMP [76]. Conversely, the PmB can exert a pro-biofilm effect as recently reported for *Acinetobacter baumanii* [77]. Regarding *V*. *cholerae*, a recent study demonstrated that the cranberry extract can inhibit the biofilm formation at the maturation stage by inhibiting the c-di-GMP pathway. In addition, a study from Rasmussen and collaborators identified a promising inhibitor of the sodium-driven flagellar motor of *V*. *cholerae* [78], which is important for biofilm formation [71]. Interestingly, this chemical inhibitor can also reduce the expression of cholera toxin and toxin-coregulated pilus, the main virulence factors of *V*. *cholerae*, and decrease biofilm formation and fluid secretion in a rabbit ileal loop model [78]. Here we demonstrated that sub-lethal concentration of PmB can inhibit the biofilm formation in *V*. *cholerae* at the initial adhesion stage. Our results also suggest that the maturation stage of the biofilm formation is not affected by a PmB supplementation since no difference, or even a slight but not significant increase, in biofilm formation can be observed when PmB is added on preformed biofilm. Since the flagella is essential for the initiation of the biofilm formation, we hypothesize that the flagella loss is responsible for the drastic biofilm reduction observed at the initial stage of biofilm formation in presence of sub-lethal concentration of PmB. Therefore, as proposed for other antimicrobial peptides with strong anti-biofilm activity [79], PmB might represent a good candidate for the prevention of biofilm formation.

## Supporting information

S1 Fig Motility reduction is dose-dependent. Aliquots of A1552 in exponential phase of growth were dropped in the center of a Petri dish containing LB agar 0.3% and decreasing concentration of PmB or colistin (12.5μg/mL to 0.39μg/mL). A control without AMP is shown on the right (0). Photographs were taken after 24h incubation at 37°C and are representative of 3 independent experiments.
(TIFF)

S2 Fig The supernatant fraction is not contaminated with cytoplasmic proteins. Immunoblot analysis of the cytoplasmic cyclic AMP receptor protein (CRP) evaluated using-CRP antibody in the pellets and in supernatant of A1552 and MO10 in absence (-) and presence (+) of 25 μg/mL PmB. P: Pellet, S: Supernantant.
(TIFF)

S3 Fig Sub-inhibitory concentration of PmB does not alter the cell wall integrity. *V*. *cholerae* A1552 (A) and MO10 (B) cell-wall integrity in presence (black and red) in absence (gray and blue) of 25μg/mL of PmB appreciated via flow cytometry using propidium iodide probe (PI). PI fluorescence is correlated with envelope impaired cells. A positive control of pore formation (orange) consisting on a short incubation of the bacteria with high concentration of PmB (1 mg/mL) has been performed in parallel. Cell population was primarily selected in SSC/FSC then DAPI positive strains were selected. The positive control was used to set up the PI positive (PI+) and negative (PI-) threshold. Number of subpopulation events ≥ 35000.
(TIFF)

S4 Fig Colony forming units per milliliter in exponential phase. V. cholerae A1552 and MO10 CFU/mL in absence (white and blue) and in presence (black and red) of 25μg/mL of PmB after 2 hours final culture. Slight but no significant decrease in CFU/mL were observed in presence of PmB in mid-exponential phase.
(TIFF)

S1 Table Effects of polymyxin B on A1552 and MO10 on proportion of flagellated cells and flagella aspect^a^. ^a^Effects of PmB on A1552 and MO10 cells proportion having flagellum was evaluated in electron microscopy. Aspect of flagella, misshapen (bulb-like structure) or membrane loss, has also been evaluated. Cells were incubated with 25 μg/mL PmB (+PmB) or without PmB (Ø PmB), samples were taken in mid-exponential phase and treated for electron microscopy acquisitions. For each strain and conditions ≥ 170 cells were counted for presence or absence of flagella and ≥ 63 flagella for flagella aspect.
(DOCX)
