# Supplementary figures and images for: Flagella-dependent inhibition of biofilm formation by sub-inhibitory concentration of polymyxin B in Vibrio cholerae
Source: PLoS One. 2019 Aug 20;14(8):e0221431. doi: 10.1371/journal.pone.0221431 (PMC6701800; doi:10.1371/journal.pone.0221431)

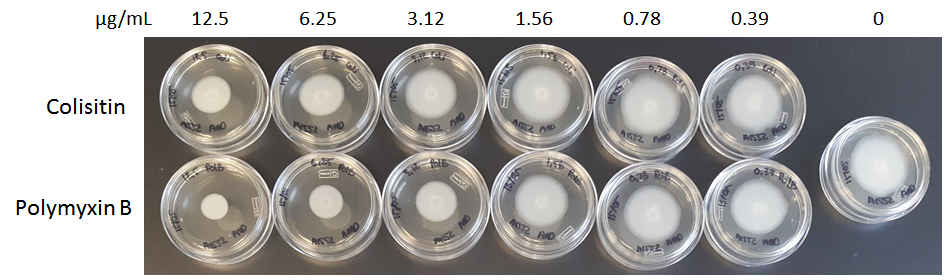

Supplement: S1 Fig — Aliquots of A1552 in exponential phase of growth were dropped in the center of a Petri dish containing LB agar 0.3% and decreasing concentration of PmB or colistin (12.5μg/mL to 0.39μg/mL). A control without AMP is shown on the right (0). Photographs were taken after 24h incubation at 37°C and are representative of 3 independent experiments. (TIFF) [file pone.0221431.s001.tiff]

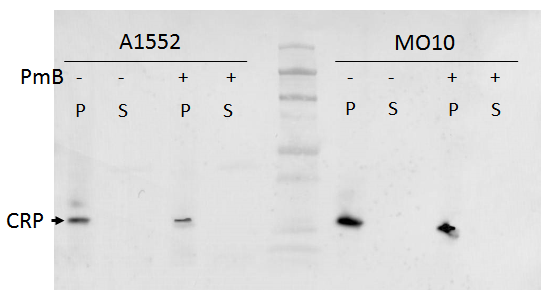

Supplement: S2 Fig — Immunoblot analysis of the cytoplasmic cyclic AMP receptor protein (CRP) evaluated using-CRP antibody in the pellets and in supernatant of A1552 and MO10 in absence (-) and presence (+) of 25 μg/mL PmB. P: Pellet, S: Supernantant. (TIFF) [file pone.0221431.s002.tiff]

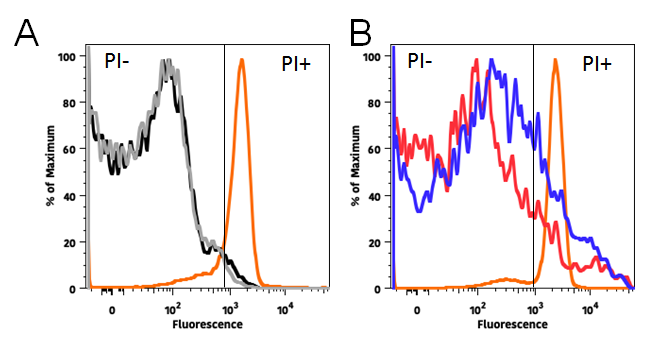

Supplement: S3 Fig — V. cholerae A1552 (A) and MO10 (B) cell-wall integrity in presence (black and red) in absence (gray and blue) of 25μg/mL of PmB appreciated via flow cytometry using propidium iodide probe (PI). PI fluorescence is correlated with envelope impaired cells. A positive control of pore formation (orange) consisting on a short incubation of the bacteria with high concentration of PmB (1 mg/mL) has been performed in parallel. Cell population was primarily selected in SSC/FSC then DAPI positive strains were selected. The positive control was used to set up the PI positive (PI+) and negative (PI-) threshold. Number of subpopulation events ≥ 35000. (TIFF) [file pone.0221431.s003.tiff]

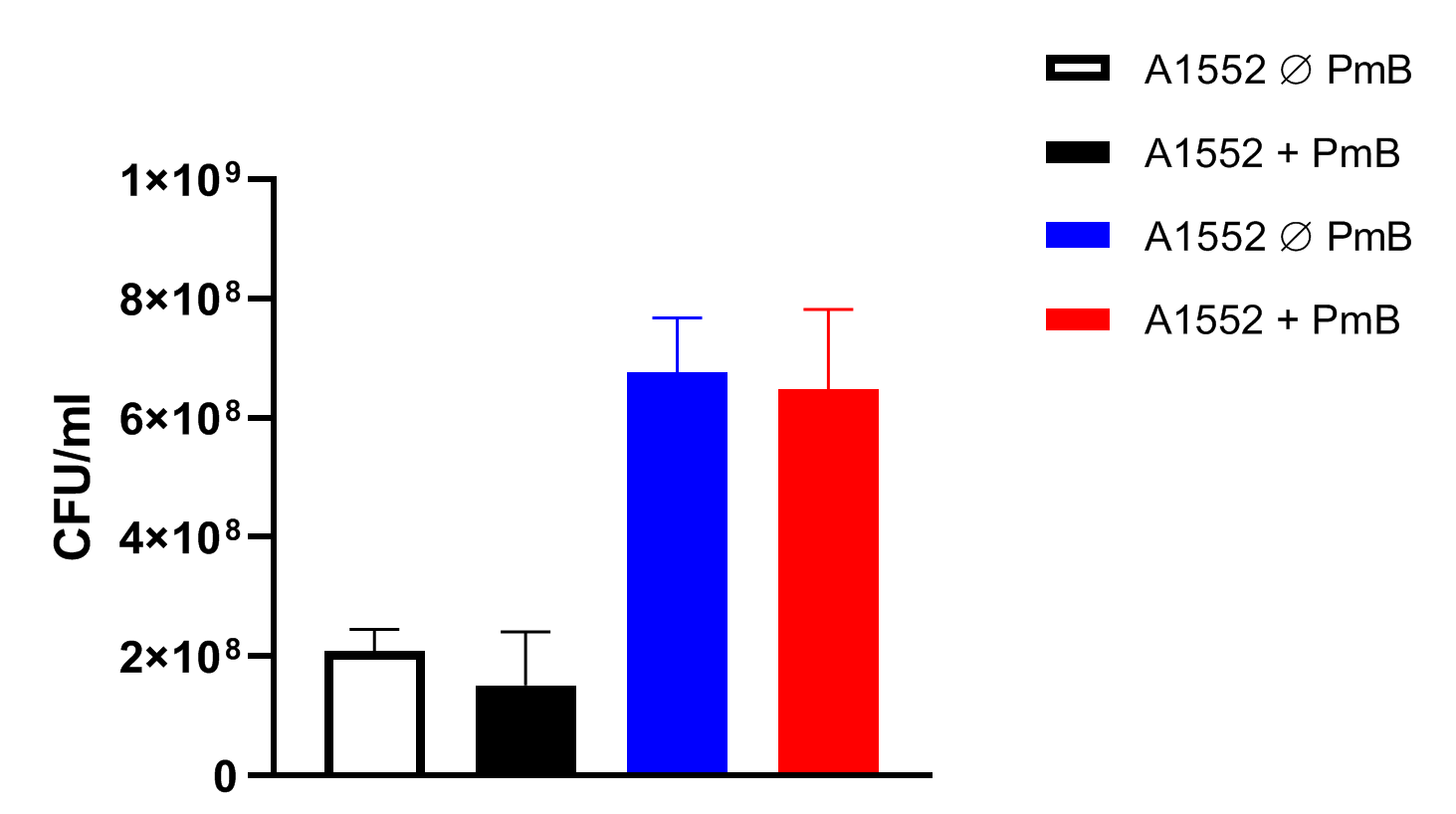

Supplement: S4 Fig — Slight but no significant decrease in CFU/mL were observed in presence of PmB in mid-exponential phase. (TIFF) [file pone.0221431.s004.tiff]
