# Supplementary material for: Flagella-dependent inhibition of biofilm formation by sub-inhibitory concentration of polymyxin B in Vibrio cholerae
Source: PLoS One. 2019 Aug 20;14(8):e0221431. doi: 10.1371/journal.pone.0221431 (PMC6701800; doi:10.1371/journal.pone.0221431)
Supplement: S1 Table — aEffects of PmB on A1552 and MO10 cells proportion having flagellum was evaluated in electron microscopy. Aspect of flagella, misshapen (bulb-like structure) or membrane loss, has also been evaluated. Cells were incubated with 25 μg/mL PmB (+PmB) or without PmB (Ø PmB), samples were taken in mid-exponential phase and treated for electron microscopy acquisitions. For each strain and conditions ≥ 170 cells were counted for presence or absence of flagella and ≥ 63 flagella for flagella aspect. (DOCX) [file pone.0221431.s005.docx]

|  | |  | **Flagellated bacteria** | **Misshapen or uncoated flagella** | **Flagella with bulb** |
| --- | --- | --- | --- | --- | --- |
| **A1552** | **Ø PmB** |  | 44,6% | 5,4% | 2,0% |
|  | **+ PmB** |  | 19,1% | 61,4% | 50,6% |
|  |  | **Relative change +PmB / ØPmB** | -57,1% | +1036,7% | +2396,4% |
| **MO10** | **Ø PmB** |  | 51,2% | 11,1% | 9,5% |
|  | **+PmB** |  | 45,1% | 46,1% | 43,3% |
|  |  | **Relative change +PmB / ØPmB** | -11,9% | +315,0% | +355,0% |
